# α-Tocopherol and β-carotene concentrations in feed, colostrum, cow and calf serum in Swedish dairy herds with high or low calf mortality

**DOI:** 10.1186/s13028-018-0361-0

**Published:** 2018-02-01

**Authors:** Maria Torsein, Ann Lindberg, Catarina Svensson, Sören Krogh Jensen, Charlotte Berg, Karin Persson Waller

**Affiliations:** 1 Farm & Animal Health, Uddetorp Röda huset, 532 96 Skara, Sweden; 2 Department of Animal Environment and Health, Swedish University of Agricultural Sciences, P.O. Box 234, 532 23 Skara, Sweden; 3 Department of Disease Control and Epidemiology, National Veterinary Institute, 751 89 Uppsala, Sweden; 4 Department of Clinical Sciences, Swedish University of Agricultural Sciences, P.O. Box 7054, 75007 Uppsala, Sweden; 5 Department of Animal Science, Aarhus University, Research Centre Foulum, P.O. Box 50, 8830 Tjele, Denmark; 6 Department of Animal Health and Antimicrobial Strategies, National Veterinary Institute, 751 89 Uppsala, Sweden

**Keywords:** Calf mortality, Colostrum, Transition milk, Vitamin A, Vitamin E

## Abstract

**Background:**

A study of herd-level risk factors for calf mortality in large Swedish dairy herds showed low serum concentrations of α-tocopherol and β-carotene in 1–7 day old calves to be more common in high mortality herds. Therefore, we aimed to investigate if calf mortality risk at herd level is associated with concentrations of α-tocopherol and/or β-carotene at individual level in feed, colostrum, cow and calf serum, while controlling for herd level covariates. Inclusion criteria were affiliation to the Swedish official milk recording scheme, herd size of ≥ 120 milking cows/year, calf mortality risk (day 1–90) of at least 6% (high mortality; HM) or less than 1% (low mortality; LM) and located within one of two regions in southern Sweden. This cross-sectional study was performed in 2010 in 19 (n_HM_ = 9; n_LM_ = 10) dairy herds. Questionnaires were used to collect information about feed and routines for colostrum feeding. Feed (n = 57), colostrum (n = 162), cow serum (n = 189) and calf serum samples (n = 187) were collected and analysed for α-tocopherol and β-carotene. Other analyses e.g. total serum protein, fat content, and total solids in colostrum were also performed. Linear regression models with vitamin concentrations in feed, colostrum, cow and calf serum as outcome were performed.

**Results:**

Calves in HM herds had lower concentrations of α-tocopherol in serum than calves in LM herds, but the effect depended on total protein status in serum of the calf (P = 0.036). Calves from herds that fed transition milk for 3 days or more had higher α-tocopherol concentrations in serum than calves from herds feeding transition milk up to 2 days (P = 0.013). Fat percentage in colostrum was positively associated with α-tocopherol (P < 0.001) and β-carotene concentrations in colostrum (P < 0.001). A diet containing ≥ 20% (in kg dry matter) maize silage of the total ration was negatively associated with β-carotene concentration in cow serum (P = 0.001).

**Conclusions:**

High calf mortality risks were associated with lower concentrations of α-tocopherol in calf serum for calves with failure of passive transfer. Feeding transition milk longer was associated with higher concentrations of α-tocopherol in calf serum. In HM herds, evaluation of the calves’ α-tocopherol status is recommended.

**Electronic supplementary material:**

The online version of this article (10.1186/s13028-018-0361-0) contains supplementary material, which is available to authorized users.

## Background

Calf mortality is complex and often multi-factorial in its nature, at the same time it is an economic and ethical burden for the cattle industry. For these reasons, it is important to determine factors influencing calf mortality. In a previous study of herd-level risk factors for calf mortality in large Swedish dairy herds [1], low serum concentrations of α-tocopherol (vitamin E) and β-carotene in 1–7 day old calves were found to be more common in high mortality (HM) herds than in low mortality (LM) herds.

Vitamin E comprises eight fat soluble compounds, tocopherols and tocotriols, of which α-tocopherol has the highest activity and is the major tocopherol found in blood and milk in cows [2]. Vitamin E is an antioxidant and thereby protects cells from oxidative damage. Several studies have reported that the serum α-tocopherol concentration in calves is important for their immune system [3–5]. Furthermore, Carter et al. [6] demonstrated that calves with respiratory tract disease supplemented with α-tocopherol had lower treatment costs compared to non-supplemented calves indicating a quicker recovery. Moreover, α-tocopherol has been shown to exert a protective effect on leukocytes participating in the defence against *Mannheimia haemolytica* [7].

Among carotenoids, β-carotene is the main dietary precursor of vitamin A (retinol) in cattle [8]. β-Carotene can be degraded to higher or lower extent in the rumen due to differences between the carotenoid supplement form. β-Carotenes in forage passes the rumen, i.e. have been shown to be degraded to a lower extent, whereas carotenoids supplemented as purified products have been shown to be degraded to a higher extent [8]. β-Carotenes can be converted into vitamin A in numerous cell types, but this occurs mainly in enterocytes and hepatocytes [8]. Absorbed β-carotene is transported with lipids to the liver [9, 10]. β-Carotene is an antioxidant and has immune regulatory properties [11]. A deficiency could lead to a higher risk of infections, exemplified by the increased risk of diarrhoea in calves seen during the first week after birth when the dam has inadequate β-carotene concentration in colostrum, or if the serum concentrations of the calves are low [12–14].

Only negligible amounts of α-tocopherol and β-carotene are transferred from cow to calf via the placenta [15–17], and hence colostrum is the major vitamin source for the newborn calf. Adequate colostrum intake from cows fed adequate amounts of vitamins is therefore essential for supplying calves with fat-soluble vitamins. Roughage is the cows major natural source for α-tocopherol and β-carotene, but it is difficult to predict vitamin concentrations in the roughage, as it is highly variable depending on plant species, maturity of the plant, and harvest conditions [18]. Moreover, the vitamin concentration in silage often decreases during storage [19]. Vitamins can also be supplemented in the cow’s diet, which can impact the vitamin status of the cow. Biological and genetic factors of the cow, and the cow’s dry matter intake at the time around calving are other factors that affect the concentration of vitamins in colostrum [20, 21]. Hence, the vitamin concentration in colostrum, as well as the vitamin status of the calves, is often unknown.

To evaluate if vitamin status is associated with calf mortality, more knowledge on associations between feed vitamin status, cow vitamin status, colostrum feeding and calf vitamin status is needed. Therefore, we aimed to evaluate concentrations of α-tocopherol and β-carotene in feed, colostrum and cow and calf serum at individual/sample level to test the hypothesis that calf mortality risk at herd level is associated with low average serum concentrations of α-tocopherol and/or β-carotene at individual/sample level, while controlling for other individual/sample and herd level covariates.

## Methods

### Study design

This cross-sectional study was performed in 19 dairy herds from the south of Sweden from January 14th to April 1st, 2010. As previously mentioned, we aimed to evaluate concentrations of α-tocopherol and β-carotene in HM and LM herds. Therefore, for each of the two vitamins, α-tocopherol and β-carotene, we investigated four different outcomes in the chain of events: vitamin concentrations in feed, colostrum and cow and calf serum at the individual or sample level.

### Selection of herds

The Swedish official milk recording scheme (SOMRS) produces its annual statistics based on periods ranging from September 1st up to and including August 31st the following year. This time period is used hereafter and referred to as 2008/09, 2009/10, etc. Since SOMRS data on herd size and calf mortality risks were intended to be used for the selection of the herds, the primary inclusion criteria were consequently set to be affiliation to SOMRS. Secondly the herds were selected based on geographic region, their herd size between 2006/2007 and 2008/2009 (≥ 120 cows), and their calf mortality risk in 1–90 days old calves 2008/2009. For logistic reasons herds from two regions in southern Sweden were selected, the former county of Skaraborg and south of Älvsborg (hereafter referred to as Skaraborg), and the county of Halland together with the eastern part of the county Skåne (Additional file 1). With the hypothesis that low vitamin concentrations contribute to calf mortality, we selected herds that were highly different in calf mortality to generate a study population where risk factors contributing to the variation in vitamin status would be more easily identified. Therefore, we included herds with a calf mortality risk (2008/09) of at least 6% (High Mortality, HM) or less than 1% (Low Mortality, LM).

Herds conforming to the inclusion criteria were contacted by mail and by phone, until five HM herds and five LM herds from each of the two regions had agreed to participate. One HM herd from Skaraborg was excluded from the study due to the diagnosis of Q-fever in the herd. A replacement herd was not included because the study had already started. Hence, 19 herds participated in the study, 10 LM herds and nine HM herds.

### Collection of data

As previously mentioned, data on herd size, calf mortality, and milk production were obtained from the SOMRS. During the study period January 14th, 2010 to April 1st, 2010 each herd was visited by the same veterinarian (the first author) three to five times for collection of questionnaires, information on feeding routines for individual calves and samples.

#### Questionnaire

Upon the first visit, a questionnaire was used to collect information about the feeding of cows and heifers close to calving and about routines for feeding colostrum to calves. The questionnaire had been reviewed by two veterinarians being specialists in ruminant medicine prior to the study. It comprised eight semi-closed or open questions (Additional file 2), and took approximately 15 min to complete. The farmers completed the questionnaire independently. It included questions about feeding routines and the rations of the roughage of dry cows and heifers near calving, including how minerals and vitamins were given to heifers and cows 1 month before calving and around the time of calving. This information was used to create three variables; “percentage grass silage of total diet (DM)”, “percentage maize silage of total diet (DM)”, way of feeding supplements”. Information about the general herd-level strategies for colostrum management, for example way of feeding colostrum, was also collected from the questionnaire and used to create the variable “herd-level percentage calves given colostrum manually” (i.e. fed by the farmer).

#### Data regarding feeding routines for individual calves

The farmers were instructed to fill in individual forms (Additional file 3) for up to ten calves per herd concerning the feeding routines during the calves’ first 7 days of life, i.e. what type of milk source that was fed and how long the calf stayed with their mother. For practical reasons information was not recorded specifically for calves subsequently blood sampled by the veterinarian. The information from the forms was used to create two herd-level variables: “herd-level median volume of the first colostrum meal given manually to each calf” and “herd-level median total volume of colostrum given manually the first day postpartum to each calf”.

### Sampling and analyses of samples

#### Handling of collected samples

Samples collected at the herd visits by the veterinarian (feed, blood and bulk milk) were kept cool in a cooler box, whereas colostrum samples collected by the farmers were kept cool in a refrigerator until the samples were sent by mail to the National Veterinary Institute (SVA) in Uppsala, Sweden, on the day of collection. If collected on a Friday or a Saturday the samples were refrigerated at 4 °C until sent, to reach the laboratory on a weekday.

#### Feed samples

At each visit, one pooled sample per herd of the roughage in the diet of dry cows and heifers near calving, i.e. grass silage or total mixed ration (TMR), was collected, resulting in a total of maximum three samples per herd (n = 57 samples; 27 from HM herds and 30 from LM herds). For each sample, information about when the roughage had been harvested and how it had been stored until feeding was included. This information was used to form eight variables: “first-harvest grass in feed sample” (0/1), “second-harvest grass in feed sample” (0/1), “third-harvest grass in feed sample” (0/1), “TMR” (0/1) “Region” (Halland/former Skaraborg), “Grass stored as round bales” 0/1), “Grass stored in bunker silos” (0/1), “Grass stored in tower silos” (0/1). To get a representative pooled sample, ten subsamples were collected from different parts of the feed bunk, of the dry cows or heifers near calving. The subsamples were pooled in a big plastic bag and mixed thoroughly, and approximately 1.5 L of the pooled feed sample was transferred into a new clean plastic bag. Then, air was evacuated and the bag was closed. At SVA, feed samples were stored at − 20 °C, until they later were sent to Aarhus University, Department of Animal Science, Research Center Foulum, Tjele, Denmark (RCF) and analysed for concentrations of α-tocopherol and β-carotene as described by Müller et al. [22].

#### Colostrum samples

Upon the first visit by the veterinarian, the farmers were provided with materials and written instructions for sampling colostrum from up to 10 cows per herd. All colostrum samples (178 samples from 19 herds; 83 HM herds and 95 LM herds) were collected by the farmers. The farmers were instructed to hand-milk equal amounts from each udder quarter into a clean cup (containing 40 mL), stir the sample with a disposable spoon and then pour the mixed sample into two sterile 10 mL tubes without additives and also into one 50 mL tube containing 50 µL of 20% bronopol and 0.2% methylene blue. Samples were to be collected by the farmers within 6 h post-partum and to be kept cool in a refrigerator until they were sent by mail to SVA, as described above. They were also asked to fill in a form about sampling date, time after calving when the sample was taken, parity and breed of the sampled cows and information on whether milk leakage had occurred (definition: milk puddle more than 15 cm in diameter) during the last 14 days pre-partum. For practical reasons, it was not possible to take colostrum samples from the same cows that were blood sampled.

Colostrum samples that were older than 7 days on their arrival at SVA were excluded from analysis. Of the 178 colostrum samples, 11 samples (3 HM and 8 LM) were later omitted due to blood content in colostrum. At SVA, fresh colostrum samples were analysed for contents of total solids using the Brix scale, as an indicator of immunoglobulin contents [23], with a digital refractometer (PAL-1, Atago Co., Ltd., Tokyo, Japan). Furthermore, 162 of the 167 fresh colostrum samples (78 HM and 84 LM samples) were analysed for fat, protein and lactose contents by mid-infrared spectroscopy (Fourier Transform Instrument, FT 120, Foss Electric) at the Swedish University of Agricultural Sciences (SLU), Department of Animal Nutrition and Management, Uppsala, Sweden. Consequently, five samples of fresh colostrum could not be analysed for fat, protein and lactose due to too high viscosity. Aliquots of colostrum samples were stored at SVA at − 20 °C, until they later were sent to RCF and analysed for concentrations of α-tocopherol and β-carotene as described by Jensen and Nielsen [24].

#### Blood samples of cows and calves

At each visit, the veterinarian collected blood samples from cows at 2–7 days post calving and calves at 2–7 days of age, up to a maximum total number of 10 samples from cows and 10 samples from calves per herd. When possible, mother–offspring pairs were sampled. Blood samples were collected from the coccygeal vessels of selected cows (189 samples from 19 herds; 90 HM and 99 LM), and from the jugular vein of selected calves (187 samples from 19 herds; 89 HM and 98 LM) in vacuumed tubes without additives. Altogether, 164 mother–offspring pairs were sampled from the herds (79 HM and 85 LM pairs), i.e. 87 and 88% of the collected samples from cows and calves, respectively, were paired samples. Information on parity number and breed of the sampled animals was also collected.

Upon arrival, blood samples were centrifuged and serum was collected. Serum samples were analysed for concentrations of total protein by adding 40 µL fresh sera to an optical refractometer (Leica Microsystems Inc., Buffalo, NY, US). Aliquots of serum were stored at − 20 °C, until they later were sent to RCF analysed for concentrations of α-tocopherol and β-carotene as described by Jensen and Nielsen [24].

#### Bulk milk samples

As a result of a legislated control program, salmonellosis is very rare in Swedish cattle [25]. Salmonella status was therefore only determined using one bulk milk sample per herd (n = 39; 19 from HM herds and 20 from LM herds). Bulk milk samples were collected in 10 mL sterile plastic tubes for later analysis of antibodies to *S. dublin* and *S. typhimurium* as described by Nyman et al. [25], using commercial ELISAs (PrioCheck^®^ Salmonella Ab bovine Dublin and PrioCheck^®^ Salmonella Ab bovine (O-antigens: 1, 4, 5, 9, 12), Prionics AG, Schlieren-Zurich, Switzerland).

### Data editing and statistical analyses

Data editing, descriptive statistics and multivariable analyses were performed using Stata^®^version 11 (Stata Corporation, College Station, TX, USA).

As stated previously, our outcome variables were concentrations of α-tocopherol and β-carotene in feed, colostrum, cow serum and calf serum, and analysed at sample level. Thus, eight statistical models were built. To approximate the normal distribution, the outcome variables “α-tocopherol concentration in feed”, “α-tocopherol concentration in colostrum”, “α-tocopherol concentration in calf serum”, “β-carotene concentration in feed”, “β-carotene concentration in colostrum” and “β-carotene concentration in calf serum” were transformed using the natural logarithm (ln). Linear multivariable regression, controlling for clustering within herds, was used to investigate associations between the concentration of vitamins, at individual (i.e. sample level), and the predictor variables, using Stata’s “cluster” option. Predictor variables used in the different models are given in Tables 1 and 2.

**Table 1:** Results of univariable analyses (continuous variables), tested for associations with type of herd (high mortality/low mortality)

Model/variable	High mortality			Low mortality			*P* value^a^
	25 pct.	Median	75 pct.	25 pct.	Median	75 pct.	
Model feed^b^ (n = 57)							
Herd size 2008/09	154	175	222	141	151	170	0.28
% Grass silage of total diet	27	37	42	29	41	60	0.45
Model colostrum^b^ (n = 162)							
Fat^c^ (%)	3.44	4.95	7.30	2.65	3.82	6.56	0.10
Herd size 2008/09	154	175	222	141	151	170	0.34
Total solids (Brix%)	21.2	23.4	26.1	19.9	22.4	25.1	0.20
Model cow (n = 189)							
Herd size 2008/09	154	175	222	141	151	170	0.28
% Grass silage of total diet	27	37	42	29	41	60	0.45
Model calf^b^							
Herd size 2008/09	154	175	222	141	151	170	0.28
Median herd level concentration of vitamin (α-tocopherol/β-carotene) in colostrum (µg/mL)	0.59/0.07	0.90/0.11	1.19/0.17	0.72/0.08	1.07/0.15	1.44/0.20	0.030/0.18

^a^Tested with univariable logistic regression, with cluster on herd, of its association with type of herd (high mortality/low mortality)

^b^Outcome transformed with a natural logarithm (ln) to approximate normal distribution

^c^Five missing values

**Table 2:** Results of univariable analyses (categorical variables), tested for associations with type of herd: high mortality (HM) or low mortality (LM)

Model/variable	Class	HM	LM	*P* value^a^
Model feed^b^ (n = 57)				
Feeding TMR	Yes	18	21	
	No	9	9	0.88
First harvest grass in feed sample^c^	Yes	12	15	
	No	15	12	0.64
Percentage maize silage of total diet (%)	< 20	18	24	
	≥ 20	9	6	0.26
Third harvest grass in feed sample^d^	Yes	18	16	
	No	9	11	0.74
Type of herd (HM/LM)		27	30	
Model colostrum^b^ (n = 162)				
Breed	SR	15	46	
	SH/MB	65	41	0.063
Hours post-partum sample was collected	< 1	19	28	
	> 1 ≤ 3	37	30	
	> 3–6	24	29	0.15
Milk leakage occurred pre-partum	Yes	6	13	
	No	64	74	0.25
	Missing	10	0	
Parity	1	20	22	
	2	24	34	
	3	16	13	
	4–8	20	18	0.68
Type of herd (HM/LM)		80	87	
Model cow (n = 189)				
Breed	SR	14	54	
	SH	67	39	
	MB	9	6	0.12
Day post partum cow was sampled	2–3	33	40	
	4–5	31	36	
	6–7	26	23	0.80
Feeding TMR	Yes	18	21	
	No	9	9	0.88
Parity	1	37	39	
	2	21	31	
	3–8	32	29	0.55
Percentage maize silage of total diet (DM)	Tested in model feed, see above			
Type of herd (HM/LM)		90	99	
Model calf^b^ (n = 187)				
Age of calf when sampled (days)	2–3	30	42	
	4–5	30	33	
	6–7	29	23	0.53
Breed	SR	15	52	
	SH/MB	74	46	0.052
Total protein (g/L)	≥ 55	59	58	
	< 55	30	40	0.52
Transition milk is given (days)	≥ 3	59	88	
	< 3	20	10	0.42
Type of herd (HM/LM)		89	98	

*SR* Swedish Red, *SH* Swedish Holstein, *MB* mixed breed

^a^Tested with univariable logistic regression, with cluster on herd, of its association with type of herd (HM: high mortality; LM: low mortality)

^b^Outcome transformed with a natural logarithm (*ln*) to approximate normal distribution

^c^Variable used only for the model “β-carotene concentration in feed”

^d^Variable used only for the model “α-tocopherol concentration in feed”

The hypothesized causal relationships between the outcomes and the predictor variables were outlined using causal diagrams to identify potential confounding effects and intervening variables. In the models: “α-tocopherol concentration in calf serum” and “β-carotene concentration in calf serum”, variables regarding colostrum management were not entered since they all were regarded as interveners with the variable “total protein”, measured in calf serum. Variables not considered to have intervening effects were entered in the multivariable regression models after correlations between variables were assessed. Correlations between variables qualifying for inclusion in the multivariable analyses were assessed using Spearman’s rank correlation coefficients. For the models: “α-tocopherol concentration in feed” and “β-carotene concentration in feed”, strong and significant correlations were present between three variables; first-, second- and third harvest grass in feed sample, respectively. The variables with the highest explanatory power (based on pseudo *R*^*2*^) were chosen, i.e. with α-tocopherol as outcome the variable “third-harvest grass in feed sample” (pseudo-*R*^*2*^: 0.009) and for β-carotene “first-harvest grass in feed sample” (pseudo-*R*^*2*^: 0.014), and these variables were consequently used in their respective model.

For the models “α-tocopherol concentration in colostrum” and “β-carotene concentration in colostrum” the samples containing blood were omitted due to potential bias since they had significantly higher Brix% and protein concentration compared to colostrum samples not containing blood.

Before running the multivariable models, all candidate predictor variables were evaluated with univariable regression models (with vitamin concentrations as outcomes), controlling for clustering within herd, in order to detect possible bias and to explore the variables. Hence, the univariable regression models were not used to qualify variables to the multivariable models; rather, the predictors were included in the multivariable models according to our a priori constructed causal diagram, regardless of P value. The predictor variables and some data from the variables of the collected samples were also evaluated with univariable regression with type of herd (HM/LM) as outcome, as presented in Tables 1, 2, 3, and further explored with Wilcoxon rank sum test or χ^2^ test (Chi square test) to further analyse possible associations between the variables.

**Table 3:** Distribution of variables analysed in collected samples and results of univariable analyses

Model/variable	High mortality (HM)			Low mortality (LM)			*P* value^a^
	25 pct.	Median	75 pct.	25 pct.	Median	75 pct.	
Model feed (nHM = 27; nLM = 30)							
α-Tocopherol (mg/kg DM)	30.30	46.41	70.95	44.98	63.81	75.18	0.50
β-Carotene (mg/kg DM)	15.3	28.3	35.1	20.6	36.6	42.2	0.33
Number of days from collection of sample until arrival at lab	1	1	2	1	2	3	0.75
Model colostrum (nHM = 80^b^; nLM = 87^b^)							
α-Tocopherol (µg/mL colostrum)	1.58	2.62	5.68	0.91	2.25	4.37	0.085
β-Carotene (µg/mL colostrum)	0.47	0.75	1.74	0.29	0.74	1.45	0.14
Total solids (Brix%)	21.20	23.35	26.05	19.90	22.40	25.10	0.20
Fat^c^ (%)	3.44	4.95	7.30	2.65	3.82	6.56	0.10
Lactose^c^ (%)	2.82	3.10	3.31	3.01	3.21	3.50	0.003
Protein^c^ (%)	12.39	13.65	15.48	11.07	13.02	14.77	0.21
Number of days from collection of sample until arrival at lab	1	3	5	1	3	4	0.61
Model cow (nHM = 90; nLM = 99)							
α-Tocopherol (µg/mL serum)	1.75	2.34	2.75	1.98	2.59	3.00	0.17
β-Carotene (µg/mL serum)	2.17	2.95	4.09	2.40	3.37	4.46	0.39
Number of days from collection of sample until arrival at lab	1	1	2	1	2	3	0.74
Model calf (nHM = 89; nLM = 98)							
α-Tocopherol (µg/mL serum)	0.59	0.90	1.19	0.72	1.07	1.44	0.030
β-Carotene (µg/mL serum)	0.07	0.11	0.17	0.08	0.15	0.20	0.18
Total protein (g/L)	53	57	61	53	57	62	0.77
Age (days) of the calf at sampling	3	4	6	3	4	5	0.32
Number of days from collection of sample until arrival at lab	1	1	2	1	2	4	0.77

^a^Tested with univariable logistic regression with cluster on herd of its association with type of herd (HM/LM)

^b^Samples with blood in colostrum omitted (n_HM_ = 3; n_LM_ = 8)

^c^Five missing values

The multivariable models were run using a manual stepwise backward procedure, where variables associated with the outcome at P ≤ 0.05 were retained in the model until all variables in the model were significant. All variables excluded during the reduction were then re-entered and kept if the P value was ≤ 0.05 or if the parameter estimate of another variable changed more than 20%, indicating confounding [26].

Two-way interactions (considered biologically plausible by the investigator) between variables in the reduced model were tested one by one and kept if the P value was ≤ 0.05. To control for potential bias due to a skewed distribution of breed between groups (the distribution of Swedish Red, Swedish Holstein and Mixed breed in HM herds was 16, 74 and 10%, respectively; and 55, 39 and 6%, respectively, in LM herds), we forced the variables “group (HM/LM)” and “breed” into all models, except for the two models with vitamin concentration in feed as outcomes, where “breed” was not considered to be a biologically plausible predictor. Additionally, for the two models with vitamin concentration in feed as outcomes, the variable “TMR (feeding total mixed rations/not feeding total mixed rations)” was also forced into the models to control for the fact that feed samples from TMR herds are accounting for the total ration, but only for the grass silage in non-TMR herds.

To identify potentially influential observations, plots of Pearson’s residuals (r) versus the predicted values as well as univariable kernel density estimation curves were constructed. Observations with divergent values, i.e. − 3 ≤ r ≥ 3, were considered outliers and their influence on the models were further evaluated. In the model “β-carotene concentration in cow serum” three observations showed slightly high residuals. One outlier was excluded since the concentration of vitamin was questionably high (β-carotene: 9.39 µg/mL serum) and because this observation alone made the variable “days post-partum” significant. The two other observations were kept in the model since they did not influence the model and their β-carotene concentrations were reasonable.

For each significant continuous variable in each model, we calculated the predicted effect on the concentration of fat soluble vitamins of moving from the first quartile of the predictor to the third quartile. These predicted effects are provided as examples to visualise the magnitude of the associations found in our material.

## Results

### Descriptive statistics on data collected at the individual level

Predictor variables used in the different models are, as previously mentioned, given in Tables 1 and 2. The distributions of the variables generated from the collected material (feed, colostrum, cow serum and calf serum) are given in Table 3, for HM and LM herds, respectively.

From the forms regarding feeding routines of individual calves, the following herd-level data was obtained; the median of the variable “Percentage of calves given colostrum manually” was 92% (range 22–100%) in HM herds and 100% (range 15–100%) in LM groups; the median for all herds (n = 18; 1 HM herd missing) was 100%. The volume of the first colostrum feeding given manually to individual calves varied from 0.5 to 4 L with a median value of 2.5 L for both HM and LM calves. The median total volume of colostrum given manually to individual calves the first day postpartum was 3.25 L for HM herds (range 1–8 L) and 4 L in LM herds (range 0.5–8 L). The median number of days (at herd level) that the calves were fed with transition milk were 3 days (range 2–7 days) for HM herds and 4 days (range 1.5–7 days) for LM herds.

### Descriptive statistics on data collected at the herd level

From SOMRS, herd data were collected to do the selection of herds for enrolment in the study. The median calf mortality risk (2008/09) in calves 1–90 days of age in HM herds was 9% (range 6–15%) and in LM herds 0% (range 0–0%). For herd size (2006/07; 2008/09) and annual herd milk production (in kg ECM 2008/09), see Table 4.

**Table 4:** Descriptive herd data from 19 Swedish dairy herds with high or low calf mortality risk

Variable	High mortality (n = 9)			Low mortality (n = 10)			*P* value^a^
	25 pct.	Median	75 pct.	25 pct.	Median	75 pct.	
ECM (kg)/year 2008/09	8475	9516	10,207	9265	10,234	10,409	0.24
Herd size^b^ 2006/07	153	184	226	129	134	169	0.071
Herd size^b^ 2008/09	154	175	222	141	151	170	0.28
% Herd expansion^c^	− 0.058	− 0.013	0.11	− 0.040	0.074	0.10	0.80

^a^Tested with univariable logistic regression, of associations with type of herd

^b^Cows/year

^c^Calculated as [(herd size 2008/09 − herd size 2006/07)/Herd size 2008/09]

One HM herd stored their grass silage as round bales; one LM herd stored their grass silage mainly in a bunker silo, but also as round bales, while the rest of the herds stored all their grass silage in bunker silos. Most of the herds fed a total mixed ration to the cows (70% of HM herds and 67% of LM herds), 8 of 10 herds in Halland/Skåne and 5 of 9 herds in Skaraborg. The median percentage grass in DM of the full ration was 36.3% (range 20–68.9%) and 42.1% (range 18–60%) in Halland/Skåne and Skaraborg, respectively. Herds feeding TMR had significantly (P = 0.024) higher percentage of maize silage DM of full ration (Additional file 4). The median percentage maize silage DM of the full ration was significantly higher in Halland/Skåne (P = 0.005) with 17.2% (range 0–28.7%) and 0% (range 0–29.5%) in Halland/Skåne and Skaraborg, respectively (Additional file 5). All herds fed vitamin and mineral supplements to cows and heifers before calving. All bulk milk samples analysed were negative for antibodies against *S. typhimurium* and *S. dubli*n.

### Multivariable analyses

#### α-Tocopherol

The results of the four multivariable analyses for α-tocopherol are presented in Table 5.

**Table 5:** Results from multivariable linear regression models of the associations between predictors and concentration of α-tocopherol

Model/variable	Class	α-Tocopherol concentration			
		Estimate	Robust std. err.	*P* value	95% CI
Model feed α-tocopherol^a^ (mg/kg DM; n = 57)					
Constant		3.59	0.14	< 0.001	3.29–3.90
Herd size		0.0023	0.00053	< 0.001	0.0012–0.034
Region	Halland/Skåne	Baseline	–	–	–
	Skaraborg	0.28	0.14	0.065	− 0.19 to 0.58
Third harvest in feed sample	No	Baseline	–	–	–
	Yes	− 0.36	0.12	0.006	− 0.60 to − 0.12
TMR^b^	No	Baseline	–	–	–
	Yes	− 0.80	0.12	< 0.001	− 1.07 to − 0.54
Interaction: TMR × region				0.006	
	TMR: no × Halland/Skåne	Baseline	–	–	–
	TMR: yes × Halland/Skåne	− 0.80	0.13	< 0.001	− 1.07 to − 0.53
	TMR: no × Skaraborg	0.28	0.14	0.065	− 0.19 to 0.58
	TMR: yes × Skaraborg	0.08	0.11	0.48	− 0.15 to 0.32
Type of herd^b^	LM	Baseline	–	–	–
	HM	0.054	0.11	0.615	− 0.17 to 0.28
% Grass silage of total diet		0.86	0.28	0.008	0.26–1.45
Model colostrum α-tocopherol (µg/mL)^a^ (n = 162)					
Constant		− 1.68	0.32	< 0.001	− 2.34 to − 1.02
Breed^b^	SR	Baseline	–	–	–
	SH/MB	0.45	0.12	0.001	0.21–0.69
Fat^c^ (%)		0.20	0.023	< 0.001	0.15–0.25
Herd size		0.0014	0.00046	0.006	0.00047–0.0024
Type of herd^b^	LM	Baseline	–	–	–
	HM	− 0.11	0.11	0.34	− 0.35 to 0.13
Total solids (Brix%)		0.042	0.013	0.005	0.015–0.069
Model cow α-tocopherol (µg/mL) (n = 189)					
Constant		2.77	0.18	< 0.001	2.40–3.14
Breed^b^				0.10	
	SR	Baseline	–	–	–
	SH	− 0.33	0.14	0.027	− 0.62 to − 0.04
	MB	− 0.30	0.19	0.13	− 0.70 to 0.10
Parity				0.0053	
	1	Baseline	–	–	–
	2	0.26	0.15	0.10	− 0.057 to 0.57
	3–8	− 0.32	0.13	0.028	− 0.60 to − 0.040
Type of herd^b^	LM	Baseline	–	–	–
	HM	− 0.099	0.15	0.53	− 0.42 to 0.23
Model calf serum α-tocopherol^a^ (µg/mL) (n = 187)					
Constant		0.026	0.11	0.82	− 0.20 to 0.25
Breed^b^	SR	Baseline	–	–	–
	SH/MB	0.24	0.11	0.043	− 0.0081 to 0.48
Days transition milk is fed (days)	≥ 3	Baseline	–	–	–
	< 3	− 0.21	0.076	0.013	− 0.37 to − 0.052
Total protein (g/L)	≥ 55	Baseline	–	–	–
	< 55	− 0.16	0.11	0.16	− 0.40 to 0.073
Type of herd^b^	LM	Baseline	–	–	–
	HM	− 0.17	0.14	0.219	− 0.47 to 0.12
Interaction: type of herd × total protein (g/L)				0.036	
	LM × total protein ≥ 55	Baseline	–	–	–
	LM × total protein < 55	− 0.16	0.11	0.16	− 0.40 to 0.072
	HM × total protein ≥ 55	− 0.17	0.14	0.22	− 0.47 to 0.11
	HM × total protein < 55	− 0.68	0.14	< 0.001	− 0.97 to − 0.40

*SR* Swedish Red, *SH* Swedish Holstein, MB mixed breed

^a^Outcome transformed with a natural logarithm (ln) to approximate normal distribution

^b^Forced into model

^c^Five missing values

##### Feed model

No effect of type of herd, i.e. HM/LM herd, was identified. A higher percentage of grass silage (DM) of total diet was associated with higher concentrations of α-tocopherol in the analysed feed samples (P = 0.008), corresponding to a difference of 1.30 mg/kg DM when comparing the 25th percentile [27.2% grass silage (DM) of full ration] to the 75th percentile [57.5% grass silage (DM) of full ration] (95% confidence interval 1.081–1.55). Feed samples containing grass from the third harvest of the season had lower concentrations of α-tocopherol than feed samples not containing grass from the third harvest (P = 0.006). Lower concentrations of α-tocopherol were seen in feed samples coming from herds using TMR, compared to herds not using TMR (P < 0.001). However, an interaction was observed between region and TMR (P = 0.006), implying that the difference in concentration of α-tocopherol in feed samples was only significant for herds in region Halland/Skåne. Baseline herds (herds not feeding TMR in region Halland/Skåne) had a higher concentration of α-tocopherol corresponding to a difference of 0.45 mg/kg DM in feed compared to herds feeding TMR, in the same region.

##### Colostrum model

No effect of type of herd was found. Breed was significantly associated with the concentration of α-tocopherol in colostrum (P = 0.001), with higher concentrations in Swedish Holstein and mixed breed than in Swedish Red. Colostrum with a higher content of total solids (Brix%) was associated with a higher concentration of α-tocopherol (P = 0.005), corresponding to a difference of 1.25 µg/mL colostrum (95% confidence interval 1.09–1.44), when comparing the 25th percentile (Brix 20.4%) to the 75th percentile (Brix 25.4%). A higher content of fat in colostrum was also associated with a higher concentration of α-tocopherol (P < 0.001), corresponding to a difference of 3.11 µg/mL colostrum (95% confidence interval 2.48–3.91), when comparing the 25th percentile (2.91% fat) to the 75th percentile (7.1% fat).

##### Cow model

No effect of type of herd was seen. Parity was significantly associated with α-tocopherol in serum (P = 0.005), with lower concentrations in cows in third to eight parities, as compared to first parity cows.

##### Calf model

Calves in HM herds had lower α-tocopherol serum concentrations than calves in LM herds. An interaction between total protein and type of herd (HM/LM) was observed, but was only significant for HM herds (P = 0.036). For those herds, calves with total protein < 55 g/L, i.e. failure of passive transfer (FPT), had significantly lower α-tocopherol serum concentrations, compared to baseline herds (LM and total protein ≥ 55 g/L). Swedish Holstein calves or calves of mixed breed had significantly higher α-tocopherol serum concentrations compared to Swedish Red calves (P = 0.043). Calves from herds that fed transition milk for 3 days or more had significantly higher serum concentrations of α-tocopherol (P = 0.13).

#### β-Carotene

The results of the four multivariable analyses for β-carotene are presented in Table 6.

**Table 6:** Results from multivariable linear regression models of the associations between predictors and concentration of β-carotene

Model/variable	Class	β-Carotene concentration			
		Estimate	Robust std. err.	*P* value	95% CI
Model feed β-carotene (mg/kg DM)^a^ (n = 57)					
Constant		3.50	0.10	< 0.001	3.28–3.72
First harvest in feed sample	No	Baseline	–	–	–
	Yes	0.64	0.17	0.001	0.29–1.00
Region	Halland/Skåne	Baseline	–	–	–
	Skaraborg	0.38	0.17	0.034	0.032–0.73
TMR^b^	No	Baseline	–	–	–
	Yes	− 0.99	0.14	< 0.001	− 1.28 to − 0.70
Type of herd^b^	LM	Baseline	–	–	–
	HM	− 0.15	0.17	0.38	− 0.51 to 0.20
Model colostrum β-carotene (µg/mL)^a^ (n = 162)					
Constant		− 2.81	0.38	< 0.001	− 3.6 to − 2.02
Breed	SR	Baseline	–	–	–
	SH/MB	0.69	0.25	0.013	0.16 to 1.21
Fat^c^ (%)		0.26	0.27	< 0.001	0.20 to 0.32
Interaction: fat (%) × breed	Fat^c^ (%) × breed SR	Baseline	–	–	–
	Fat^c^ (%) × breed SH/MB	− 0.11	0.036	0.005	− 0.19 to − 0.038
Total solids (Brix%)		0.048	0.016	0.007	0.015 to 0.081
Type of herd^b^	LM	Baseline	–	–	–
	HM	− 0. 62	0.16	0.69	− 0.39 to 0.26
Model cow serum β-carotene (µg/mL) (n = 189)					
Constant		4.28	0.21	< 0.001	3.85–4.71
Breed^b^				0.0003	
	SR	Baseline	–	–	–
	SH	− 1.27	0.25	< 0.001	− 1.78 to − 0.75
	MB	− 0.55	0.32	0.10	− 1.23 to 0.12
Percentage maize silage (DM) of total ration	< 20%	Baseline	–	–	–
	≥ 20%	− 1.08	0.27	0.001	− 1.66 to − 0.50
Type of herd^b^	LM	Baseline	–	–	–
	HM	0.16	0.27	0.56	− 0.41 to 0.73
Model calf serum β-carotene^a^ (µg/mL; n = 187)					
Constant		− 1.84	0.08	< 0.001	− 2.01 to − 1.67
Breed^b^	SR	Baseline	–	–	–
	SH/MB	− 0.06	0.12	0.61	− 0.31 to 0.018
Interaction: breed × total protein (g/L)			0.036		
	SR × total protein ≥ 55	Baseline	–	–	–
	SR × total protein < 55	− 0.21	0.11	0.078	− 0.44 to 0.026
	SH/MB × total protein ≥ 55	− 0.062	0.12	0.61	− 0.31 to 0.18
	SH/MB × total protein < 55	− 0.78	0.15	< 0.001	− 1.08 to − 0.047
Total protein (g/L)	≥ 55	Baseline	–	–	–
	< 55	− 0.21	0.11	0.078	− 0.44 to 0.026
Type of herd^b^	LM	Baseline	–	–	–
	HM	− 0.18	0.15	0.23	− 0.49 to 0.12

*SR* Swedish Red, *SH* Swedish Holstein, *MB* mixed breed

^a^Outcome transformed with a natural logarithm (ln) to approximate normal distribution

^b^Forced into model

^c^Five missing values

##### Feed model

No effect of type of herd was identified. The concentration of β-carotene in feed was lower in herds using TMR (P < 0.001), and in region Halland/Skåne (P = 0.034), compared to herds not using TMR and region Skaraborg, respectively. Feed samples containing grass from the first harvest of the season had higher concentration of β-carotene compared to feed samples without grass from the first harvest of the season (P = 0.001).

##### Colostrum model

No effect of type of herd was shown. A higher content of fat in colostrum was associated with a higher concentration of β-carotene (P < 0.001). However, an interaction between fat and breed was observed. That means that the effect of fat is different for the different breeds. When comparing a difference in fat content in colostrum, and taking breed into account; corresponding to the 25th and 75th percentiles; i.e. 2.91 and 7.1% fat, respectively, it was shown that Swedish Red had higher concentration (1.61 µg/mL; P = 0.038) of β-carotene in colostrum compared to Swedish Holstein or mixed breed (95% confidence interval 1.17–2.20), Although Swedish Holstein cows had higher fat content in colostrum, the interaction means that Swedish Red cows had higher β-carotene concentration per unit of fat, given the other variables in the model. Total solids (Brix%) in colostrum was positively associated with the concentration of β-carotene in colostrum (P = 0.007). A comparison between the 25th percentile (Brix 20.4%) and the 75th percentile (Brix 25.4%) showed higher β-carotene concentration corresponding to a difference of 1.27 µg/mL colostrum (95% confidence interval 1.08–1.59).

##### Cow model

No effect of type of herd was identified. Having a total ration containing more or equal to 20% maize silage DM was associated with significantly lower concentration of β-carotene in cow serum (P = 0.001). Swedish Holstein cows had significantly lower concentrations of β-carotene in serum than Swedish Red cows (P < 0.001).

##### Calf model

No effect of type of herd was found. A significant negative association between FPT and concentrations of β-carotene in calf serum was found (i.e. lower concentrations in calves with FPT). However, an interaction between breed and FPT was observed (P = 0.002). This interaction means that the effect of FPT on the concentration of β-carotene in calf serum is different for the different breeds with significantly lower concentrations of β-carotene in calves of Swedish Holstein or mixed breed calves, compared to calves of Swedish Red.

## Discussion

The evaluation of α-tocopherol and β-carotene in feed, colostrum, cow and calf serum revealed that only low α-tocopherol status at the calf level was associated with high calf mortality risk at herd level. However, the low α-tocopherol status of the calf was related to the total protein status of the calf, which is a proxy for adequate colostrum intake. Still, no differences between HM and LM herds were found regarding colostrum routines in the univariable analyses. Different routines regarding the feeding of transition milk were identified, with significant higher concentrations of α-tocopherol in calf serum from herds feeding transition milk for 3 days or more.

### Vitamins in feed

Roughage is the major natural source of α-tocopherol and β-carotene for the cow, but it is difficult to predict vitamin concentrations in the feed, as these are highly variable depending on plant species, maturity of the plant, harvest and storage conditions [18]. Influence of harvest number was shown in the feed models in our study with lower concentrations of α-tocopherol in feed samples containing grass from third harvest and higher concentrations of β-carotene in feed samples containing grass from first harvest. The results of season is in contrast to findings by Jensen et al. [27], who reported higher concentrations of α-tocopherol and β-carotene in a grass-clover mixture in late season (Aug–Oct) and lower in June as compared to the average concentration for the whole season. The reason for the difference is unknown but the vitamin concentration in grass is also highly dependent on when in the vegetative phase the grass is harvested, which might have affected the result. Such data were, however, not collected in this study. Having a high proportion of grass silage of the total ration was positively associated with α-tocopherol concentrations in the feed samples. The importance of grass silage for cow vitamin status was shown in a Danish study in which milk from cows fed grass silage had a higher concentration of α-tocopherol and β-carotene than milk from cows fed maize silage [28]. Mogensen et al. [29] also reported that increasing the proportion of maize silage at the expense of grass-clover silage decreased the milk content of β-carotene.

For both α-tocopherol and β-carotene, feed samples taken in herds feeding TMR had lower vitamin concentrations than in herds not feeding TMR, although for α-tocopherol the association was dependent on region. This was expected as grain and other protein sources are fairly low in vitamin concentration. Another possible explanation for the lower concentrations in TMR might be related to the percentage grass or maize silage (DM) used in the TMR.

As vitamins may be lost during storage, differences in vitamin concentrations could be due to different methods for storing silage. In a Swedish study, it was concluded that there is a greater risk of α-tocopherol losses in silage stored as round-bales compared to in silage stored in bunker silos [19]. However, with only two herds in the study storing their silage in round-bales, the overall effect of storage in round bales is considered negligible.

### Vitamins in colostrum

Breed was also associated with vitamin concentrations in colostrum as Swedish Holstein cows, and cows of mixed breeds had higher concentrations of α-tocopherol and β-carotene than Swedish Red cows. This indicates a role of genetics in vitamin secretion into milk as observed previously [30]. In a study by Jensen et al. [20], significant effects of sire on concentrations of both α-tocopherol and β-carotene in milk, milk fat and also for the total secretion of fat-soluble vitamins into milk were shown. In the present study, we did not instruct the farmers to empty the udder completely at milking. Hence, the first colostrum milk yield is unknown and hence, differences in vitamin concentration in colostrum between the breeds could also be an effect of dilution. However, Swedish Holstein is a breed with higher milk production and should therefore be expected to have lower vitamin concentrations due to dilution. This is not supported by our data, which actually showed the opposite situation. We believe that the risk of bias is small and that the samples taken reflect the actual vitamin concentration that the farmers give to the calves when practising their usual colostrum management routines.

The content of fat in colostrum was positively associated with the concentrations of the two vitamins in colostrum, i.e. colostrum with higher fat content also had higher vitamin concentrations. This was expected, and a logical finding, since the vitamins are fat-soluble. The content of total solids was also positively associated with the concentrations of the two vitamins in colostrum. However, the variable total solids only explained a small proportion of the variation in vitamins in colostrum (data not shown). Therefore, estimation of total solids using the method applied here (a Brix refractometer) to predict the content of vitamins is questionable. To the authors’ knowledge there is, however, no reliable cow-side test for fat content in colostrum. Hence, this could be an area for future development.

### Vitamins in cow serum

The concentrations of α-tocopherol and β-carotene measured in cow serum reflects the total ration consumed by the cow. Having a total ration containing more or equal to 20% maize silage (DM) was associated with significantly lower concentration of β-carotene in cow serum. This is in line with other studies, which have shown maize silage to be very low in vitamin content [18]. As previously mentioned, several studies have found a positive association between vitamin-rich feed and/or vitamin supplements given, and vitamin concentrations in milk, and some of these studies conclude that a vitamin-rich feed also is associated with vitamin concentrations in plasma [31, 32].

Breed was associated with β-carotene concentration in cow serum; cows of Swedish Holstein and mixed breed had lower β-carotene concentrations in cow serum than Swedish Red cows, but the opposite was found for these vitamins in colostrum. For α-tocopherol in cow serum there was only a tendency for an effect of breed with lower concentrations in Swedish Holstein cows than in Swedish Red cows. The differences between breeds are probably due to genetic factors of importance for the ability to absorb vitamins in the digestive tract, or to secrete vitamins into the udder [20]. There may also have been differences between dietary fat content in the diets, but unfortunately, we have no information on dietary fat in diets for these herds. According to Goff and Stabel [21] secretion of α-tocopherol into the udder around calving is a major reason for the decreased plasma concentration observed in cows at this point in time. In both colostrum models, Swedish Holstein was associated with higher concentrations of the fat-soluble vitamins compared to Swedish Red. As above mentioned, with significant lower or a tendency for lower concentrations of β-carotene and α-tocopherol in cow serum, respectively, it seems reasonable to hypothesise that it could be an effect of colostrogenesis. However, as the blood and colostrum samples came from different cows we cannot draw any firm conclusions regarding causality. The concentrations of α-tocopherol in cows’ serum in the present study are comparable with other Scandinavian studies [20, 33].

### Vitamins in calf serum

The bulk milk samples from all herds were free from antibodies to *Salmonella*, which means that it is unlikely that salmonellosis contributed to calf mortality in this study. We found that calves in HM herds had lower concentrations of α-tocopherol in serum during the first week after birth than calves in LM herds. This association depended, however, on the total protein content of the calf serum. This is partly in line with Torsein et al. [1], who reported that the proportion of calves with inadequate concentration of α-tocopherol and β-carotene in serum was significantly higher in herds with high calf mortality risks day 1–90. In that study, which was conducted at the herd level, correlations between inadequate levels of α-tocopherol or β-carotene and failure of passive transfer could not be identified. In the present study, however, we found that calves with FPT were associated with lower concentrations of β-carotene concentration in calf serum. However, the association depended on the breed of the calves, i.e. only in calves of Swedish Holstein or mixed breed FPT was associated with lower concentrations of β-carotene in calf serum. For α-tocopherol, however, the association depended on type of herd, i.e. calves from HM herds and with failure of passive transfer were associated with lower concentrations of α-tocopherol in calf serum. Nevertheless, in this study we were not able to find any associations between colostrum management and type of herd (HM/LM), nor detect any significant difference in total protein content in serum between HM and LM herds. The lower concentrations of α-tocopherol in calves in HM herds can therefore not be fully explained by different colostrum routines and is also likely related to the quality of the feed and feeding management. We did find that calves from herds that fed transition milk for 3 days or more had higher α-tocopherol serum concentrations than calves from herds feeding transition milk up to 2 days only. The median (herd level) number of days the calves were fed with transition milk was 3 days for HM herds and 4 days for LM herds. Transition milk is sometimes mixed with colostrum and if so, the calves are offered a milk source with presumably high vitamin content.

### Study design

Our aim was to investigate if calf mortality risk at herd level is associated with concentrations of α-tocopherol and/or β-carotene at individual level in feed, colostrum, cow and calf serum. A benefit of modelling the concentrations in separate models is that intervening between variables that lie along the same causal pathway is avoided. Since this study did not aim to follow how the vitamin uptake from the feed correlates to vitamin concentration in the cow and, then to the vitamin concentration in colostrum and later concentration in the calf at individual level, we did not focus on getting samples from that chain since the evaluation of the previous step in the causal chain were at herd level in the models. Still, a high percentage of the samples from cows and calves were indeed mother/offspring pairs. However, our primary aim was to look at vitamin concentrations at different steps and evaluate them as an indicator for the herds’ vitamin status. Because of this, some associations may have remained undetected.

## Conclusions

Our hypothesis that inadequate α-tocopherol and β-carotene concentrations in calf serum were associated with high calf mortality risks could only be confirmed for α-tocopherol, but the effect depended on total protein status of the calf. In herds with high calf mortality, evaluation of the calves’ α-tocopherol status is recommended. Feeding transition milk for 3 days or more was associated with higher α-tocopherol concentrations in calf serum. High proportions of maize silage (DM) in the total diet were associated with lower concentrations of β-carotene in cow serum. Breed was associated with vitamin concentrations in colostrum.

## Additional files

**Additional file 1.** Map of Sweden showing the 19 herds in the study; 9 herds with high calf mortality (HM) and 10 herds with low calf mortality (LM). Black: county of Skaraborg and south of Älvsborg. Grey: County of Halland. Blue: County of Skåne. Red: High mortality herds. Green: Low mortality herds.

**Additional file 2.** Vitamin E and beta carotene in cow/calf/colostrum. Questionnaire.

**Additional file 3.** Forms for individual calves regarding feeding routines.

**Additional file 4.** Differences of proportion of maize in 19 Swedish dairy herds with high (n = 9) or low (n = 10) calf mortality risk, day 1–90, feeding total mixed ration (TMR) or not.

**Additional file 5.** Regional differences concerning proportion of maize of total diet in 19 Swedish dairy herds with high (n = 9) or low (n = 10) calf mortality risk, day 1–90.

## Abbreviations

DM: dry matter
FPT: failure of passive transfer, total protein < 55 g/L
HM: herd with high calf mortality risk
LM: herd with low calf mortality risk
SOMRS: Swedish Official Milk Recording Scheme
SVA: National Veterinary Institute, Uppsala, Sweden
RCF: Aarhus University, Department of Animal Science, Research Center Foulum, Tjele, Denmark

## References

[1] Torsein M, Lindberg A, Sandgren CH, Waller KP, Törnquist M, Svensson C (2011). Risk factors for calf mortality in large Swedish dairy herds. Prev Vet Med.

[2] Pehrson B, Hakkarainen J (1986). Vitamin E status of healthy Swedish cattle. Acta Vet Scand.

[3] Reddy PG, Morrill JL, Minocha HC, Morrill MB, Dayton AD, Frey RA (1986). Effect of supplemental vitamin E on the immune system of calves. J Dairy Sci.

[4] Rajaraman V, Nonnecke BJ, Franklin ST, Hammell DC, Horst RL (1998). Effect of vitamins A and E on nitric oxide production by blood mononuclear leukocytes from neonatal calves fed milk replacer. J Dairy Sci.

[5] Higuchi H, Nagahata H (2000). Effects of vitamins A and E on superoxide production and intracellular signalling of neutrophils in Holstein calves. Can Vet J.

[6] Carter JN, Gill DR, Krehbiel CR, Confer AW, Smith RA, Lalman DL (2005). Vitamin E supplementation of newly arrived feedlot calves. J Anim Sci.

[7] Urban-Chmiel R, Hola P, Lisiecka U, Wernicki A, Puchalski A, Dec M (2011). An evaluation of the effects of α-tocopherol and ascorbic acid in bovine respiratory disease complex occurring in feedlot calves after transport. Livest Sci.

[8] Nozière P, Graulet B, Lucas A, Martin B, Grolier P, Doreau M (2006). Carotenoids for ruminants: from forages to dairy products. Anim Feed Sci Technol.

[9] Chew BP (1987). Vitamin A and β-carotene on host defense. J Dairy Sci.

[10] Rizzo A, Ceci E, Pantaleo M, Mutinati M, Spedicato M, Minoia G (2013). Evaluation of blood and milk oxidative status during early postpartum of dairy cows. Animal.

[11] Chew BP (1993). Role of carotenoids in the immune response. J Dairy Sci.

[12] Lotthammer KH (1979). Importance of β-carotene for the fertility of dairy cattle. Feedstuffs.

[13] Puls R (1994). Vitamin levels in animal health. Diagnostic data and bibliographies.

[14] Kume S, Toharmat T (2001). Effect of colostral β-carotene and vitamin A on vitamin and health status of newborn calves. Livest Prod Sci.

[15] Van Saun RJ, Herdt TH, Stowe HD (1989). Maternal and fetal vitamin E concentrations and selenium–vitamin E interrelationships in dairy cattle. J Nutr.

[16] Nonnecke BJ, Horst RL, Waters WR, Dubeski P, Harp JA (1999). Modulation of fat-soluble vitamin concentrations and blood mononuclear leukocyte populations in milk replacer-fed calves by dietary vitamin A and β-carotene. J Dairy Sci.

[17] Zanker IA, Hammon HM, Blum JW (2000). β-Carotene, retinol and α-tocopherol status in calves fed the first colostrum at 0–2, 6–7, 12–13 or 24–25 hours after birth. Int J Vitam Res.

[18] Mogensen L, Kristensen T, Søegaard K, Jensen SK, Sehested J (2012). Alfa-tocopherol and beta-carotene in roughages and milk in organic dairy herds. Livest Sci.

[19] Nadeau E, Johansson B, Jensen SK, Olsson G. Vitamin content of forages as influenced by harvest and ensiling techniques. In: Grassland science in Europe, vol. 9. Zürich: vdf Hochschulverlag AG an der ETH Zurich; 2004. p. 891–3.

[20] Jensen SK, Johannsen AKB, Hermansen JE (1999). Quantitative secretion and maximal secretion capacity of retinol, β-carotene and α-tocopherol into cows’ milk. J Dairy Res.

[21] Goff J, Stabel J (1990). Decreased plasma retinol, α-tocopherol, and zinc concentration during the periparturient period: effect of milk fever. J Dairy Sci.

[22] Muller CE, Moller J, Jensen SK, Uden P (2007). Tocopherol and carotenoid levels in baled silage and haylage in relation to horse requirements. Anim Feed Sci Technol.

[23] Bielmann V, Gillan J, Perkins NR, Skidmore AL, Godden S, Leslie KE (2010). An evaluation of Brix refractometry instruments for measurement of colostrum quality in dairy cattle. J Dairy Sci.

[24] Jensen SK, Nielsen KN (1996). Tocopherols, retinol, β-carotene and fatty acids in fat globule membrane and fat globule core in cows’ milk. J Dairy Res.

[25] Nyman A-K, Agren E, Bergstrom K, Wahlstrom H (2013). Evaluation of the specificity of three enzyme-linked immunosorbent assays for detection of antibodies against Salmonella in bovine bulk milk. Acta Vet Scand.

[26] Dohoo IR, Wayne M, Stryhn H (2009). Veterinary epidemiologic research.

[27] Jensen SK, Søegaard K, Sehested J, Lindqvist H, Nadeau E (2010). Indflydelse af høstmetode og konservering på vitamin-og fedtsyreindhold. Intern Rep Husb.

[28] Havemose MS, Weisbjerg MR, Bredie WLP, Nielsen JH (2004). Influence of feeding different types of roughage on the oxidative stability of milk. Int Dairy J.

[29] Mogensen L, Vestergaard JS, Fretté X, Lund P, Weisbjerg MR, Kristensen T (2010). Effect of toasting field beans and of grass-clover: maize silage ratio on milk production, milk composition and sensory quality of milk. Livest Sci.

[30] Ramalho HMM, Santos J, Casal S, Alves MR, Oliveira MBPP (2012). Fat-soluble vitamin (A, D, E, and β-carotene) contents from a Portuguese autochthonous cow breed—Minhota. J Dairy Sci.

[31] Kaewlamun W, Okouyi M, Humblot P, Remy D, Techakumphu M, Duvaux-Ponter C (2011). The influence of a supplement of β-carotene given during the dry period to dairy cows on colostrum quality, and β-carotene status, metabolites and hormones in newborn calves. Anim Feed Sci Technol.

[32] Weiss WP, Hogan JS, Wyatt DJ (2009). Relative bioavailability of all-rac and RRR vitamin E based on neutrophil function and total α-tocopherol and isomer concentrations in periparturient dairy cows and their calves. J Dairy Sci.

[33] Meglia GE, Jensen SK, Lauridsen C, Waller KP (2006). α-Tocopherol concentration and stereoisomer composition in plasma and milk from dairy cows fed natural or synthetic vitamin E around calving. J Dairy Res.

