# Supplementary material for: α-Tocopherol and β-carotene concentrations in feed, colostrum, cow and calf serum in Swedish dairy herds with high or low calf mortality
Source: Acta Vet Scand. 2018 Feb 1;60:7. doi: 10.1186/s13028-018-0361-0 (PMC5796441; doi:10.1186/s13028-018-0361-0)

Additional file 1. Map of Sweden showing the 19 herds in the study; 9 herds with high calf mortality (HM) and 10 herds with low calf mortality (LM)

Black: County of Skaraborg and south of Älvsborg
Grey: Conty of Halland
Blue: County of Skåne
Red: High mortality herds
Green: Low mortality herds

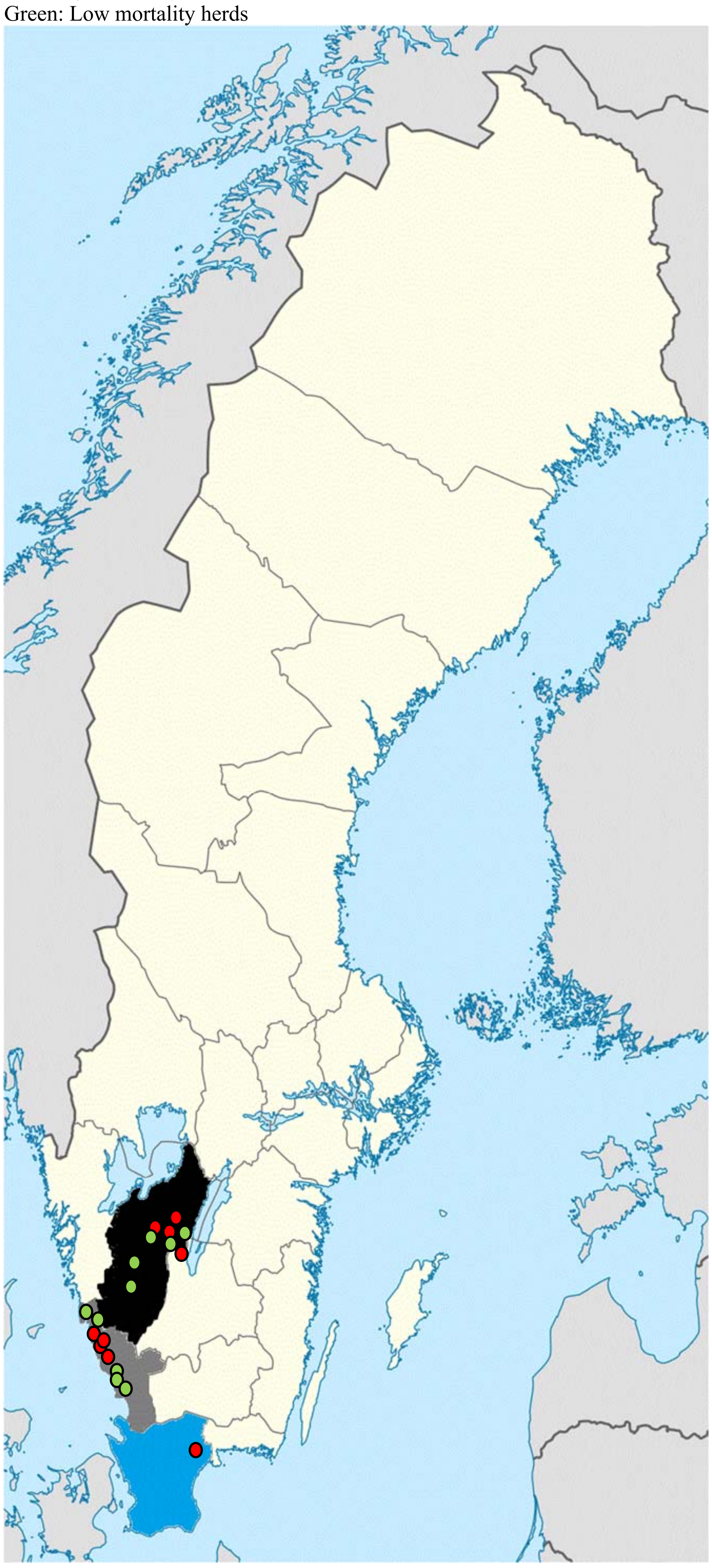

Supplement: Supplementary file 1 — Additional file 1. Map of Sweden showing the 19 herds in the study; 9 herds with high calf mortality (HM) and 10 herds with low calf mortality (LM). Black: county of Skaraborg and south of Älvsborg. Grey: County of Halland. Blue: County of Skåne. Red: High mortality herds. Green: Low mortality herds. [file 13028_2018_361_MOESM1_ESM.pdf]
