# Supplementary material for: α-Tocopherol and β-carotene concentrations in feed, colostrum, cow and calf serum in Swedish dairy herds with high or low calf mortality
Source: Acta Vet Scand. 2018 Feb 1;60:7. doi: 10.1186/s13028-018-0361-0 (PMC5796441; doi:10.1186/s13028-018-0361-0)
Supplement: Supplementary file 2 — Additional file 2. Vitamin E and beta carotene in cow/calf/colostrum. Questionnaire. [file 13028_2018_361_MOESM2_ESM.pdf]

Svenska Djurhälsovården

Additional file 2 Vitamin E and beta carotene in cow/calf/colostrum. Questionnaire.

Final version

Vitamin E and beta caroten in cow/calf/colostrum

Questionnaire

Herd: ____

The questionnaire was answered by: ________________________________

Cell phone: ___________________ Date: _________________

☐ Owner
☐ Calf caretaker/ responsible for the cattle

Questionnaire – Feeding of heifers/cows (definition:the actual feeding one month before and during the study period)

	Feeding at different time periods	Kilogram DM offered one month before calving		Around calving (± 3 days)		Space for comments
		Heifers	Cows	Heifers	Cows	
1a.)	**Roughage strategy: ☐ Total mixed ration**<br>Write how many kilograms total mixed ration (dry matter) that is offered per animal and day.<br>*NOTE! Please write the complete recipe (there is space for the recipe, see below)*					
1b.)	**Roughage strategy: ☐ Grass silage**<br>Write how many kilograms grass silage (dry matter) that is offered per animal and day.<br>*NOTE! Please write the complete recipe (there is space for the recipe, see below)*					

Space for the feed ration (recipe)

Note! Don’t forget to mention what kind of supplements that are given!
If grain or pelleted feed is bought, please write the name of the product and the manufacturer!

SVENSKA DJURHÄLSOVÅRDEN

Additional file 2 Vitamin E and beta carotene in cow/calf/colostrum. Questionnaire.

Space for the recipe of the feed ration

Vitamin/mineral supplementation	Approximately one month before calving		Around calving (± 3 days)	
	Heifers	Cows	Heifers	Cows
**2.)** Are the animals offered vitamin and mineral supplements?<br>- If yes, please answer question 2a.<br>- If no, go directly to question 3.	☐ Yes<br>☐ No	☐ Yes<br>☐ No	☐ Yes<br>☐ No	☐ Yes<br>☐ No
**2a.)** If yes on question 2- is it offered individually or in group?	☐ Individually<br>☐ In group	☐ Individually<br>☐ In group	☐ Individually<br>☐ In group	☐ Individually<br>☐ In group
**2b.)** If yes on question 2- how is it offered to the animals?	☐ Mixed into the feed<br>☐ On top of the feed<br>☐ Mineral block<br>☐ Other	☐ Mixed into the feed<br>☐ On top of the feed<br>☐ Mineral block<br>☐ Other	☐ Mixed into the feed<br>☐ On top of the feed<br>☐ Mineral block<br>☐ Other	☐ Mixed into the feed<br>☐ On top of the feed<br>☐ Mineral block<br>☐ Other
If you choose "other" in question 2b– How is it offered, please describe!				
**2c.)** If yes on question 2- what amounts of vitamin/mineral supplements are given? Write in grams per day, per animal	__________g/day/animal<br>☐ Mineral block is used	__________g/day/animal<br>☐ Mineral block is used	__________g/day/animal<br>☐ Mineral block is used	__________g/day/animal<br>☐ Mineral block is used
**2d.)** If yes on question 2– please name the manufacturer and the name of the product				

SVENSKA DJURHÄLSOVÅRDEN

Additional file 2 Vitamin E and beta carotene in cow/calf/colostrum. Questionnaire.

	Vitamin/mineral supplementation	Category	
		**Heifers**	**Cows**
**3.)**	**If No on question 2 – is vitamin/mineral supplementation offered any other time period before calving?**<br>- **If yes, please answer question 3a.**<br>- **If no, go directly to question 4.**<br><br>* Write how many days before calving	☐ Yes (When*?)<br>_____________days before calving<br>☐ No	☐ Yes (When*?)<br>_____________ days before calving<br>☐ No
**3a.)**	**If yes on question 3- is it offered individually or in group?**	☐ Individually<br>☐ In group	☐ Individually<br>☐ In group
**3b.)**	**If yes on question 3 – how is it offered?**	☐ Mixed into the feed<br>☐ On top of the feed<br>☐ Mineral block<br>☐ Other	☐ Mixed into the feed<br>☐ On top of the feed<br>☐ Mineral block<br>☐ Other
	**If you choose ”other” in question 3b–<br>How is it offered, please describe!**		
**3c.)**	**If yes on question 3- what amounts of vitamin/mineral supplements are given?**<br>- **Write in grams per day, per animal**	___________________g/day/animal<br>☐ Mineral block is used	___________________ g/day/animal<br>☐ Mineral block is used
**3d.)**	**If yes on question 3– please name the manufacturer and the name of the product**		

Calving pen

4.) Where does the main part of the calving take place?

- ☐ Calving pen, individual Number of pens:………………………
- ☐ Group calving pen for ___ cows Number of pens:………………………
- ☐ Tie stall
- ☐ Free stall
- ☐ Other:________________________________

5.) How many times does it happen that the cows are calving in the free stall or at the cubicle for tie stalls?
……..Times per year, divided by the number of calvings per year:…………………, which gives . .. …….% of the calvings per year

Colostrum management

6.) How is colostrum fed to the calf?

- ☐ The calf is always given manually (by the farmer)
- ☐ If it is clearly observed that the calf is drinking (froth around nose/mouth or swallowing loudly) it won´t get colostrum manually, but if not, it will get colostrum manually
- ☐ The calf most often suckles the dam. If it is noticed that the calf has an empty stomach, or a depressed attitude, the calf given colostrum manually
- ☐ Other...

7.) How many percent of the calves are offered colostrum manually?....................................

Calf management

8.) At what age is the calf moved to a group pen for calves? ....
