# Supplementary material for: α-Tocopherol and β-carotene concentrations in feed, colostrum, cow and calf serum in Swedish dairy herds with high or low calf mortality
Source: Acta Vet Scand. 2018 Feb 1;60:7. doi: 10.1186/s13028-018-0361-0 (PMC5796441; doi:10.1186/s13028-018-0361-0)
Supplement: Supplementary file 3 — Additional file 3. Forms for individual calves regarding feeding routines. [file 13028_2018_361_MOESM3_ESM.pdf]

SVENSKA DJURHÄLSOVÅRDEN   SVA STATENS VETERINÄRMEDICINSKA ANSTALT   SLU

Additional file 3 forms for individual calves regarding feeding routines.

CALF CARD

Identification number calf: ________________ **Date of birth:** ________________ **Time:**_______________**Breed:**____________

Identification number mother: ________________ **Breed of mother:** ________________ **Lactation number:** ________

Where was the calf born? ☐ calving pen individual ☐ group pen ☐ tie stall cubicle ☐ free stall

How many days did calf spend with mother?_____________

Herd: ________________________**Herd number (SE):** ____________

Definitions:

Colostrum = Milk from the first milking after calving
Transition milk = Milk from the second and to the 8th milking after calving
Whole milk = Milk from the cow, from later than the 8th milking.
Powder milk = All forms of milk in powder form.
Milk from cow with mastitis= the milk macroscopically changed. Please mark even if the mastitis wasn't treated with anti microbials
Water was added = If water was added to the milk, please mark it with a cross
From which individual does the milk come from = Write id nr, if many cows write the three most newly calved cows.
Disease = Write what disease or the sympthoms, i.e. arthritis, pneumonia, diarrhoea, cough and so on
Has the calf received treatment = Write any treatment, even rehydration solutions or nsaids, please also note the dose given.

	**Time**	**Volume of milk** (Liter)	**Colostrum** (Mark with X)	**Transition milk** (Mark with X)	**Whole milk** (Mark with X)	**Powder milk** (Mark with X)	**Milk from cow with mastitis** (Mark with X)	**Water was added** (Mark with X)	**From which individual does the milk come from** (id nr)	**Disease** (write symthoms or diagnose)	**Has the calf received treatment?**
**Day 1 Morning**											
**Day 1 Evening**											
**Day 2 Morning**											
**Day 2 Evening**											
**Day 3 Morning**											
**Day 3 Evening**											

Additional file 3 forms for individual calves regarding feeding routines.

CALF CARD PAGE 2

Identification number calf:_______________ Herd:_______________________________

Definitions:

Colostrum = Milk from the first milking after calving
Transition milk = Milk from the second and to the 8th milking after calving
Whole milk = Milk from the cow, from later than the 8th milking.
Powder milk = All forms of milk in powder form.
Milk from cow with mastitis= the milk macroscopically changed. Please mark even if the mastitis wasn't treated with anti microbials
Water was added = If water was added to the milk, please mark it with a cross
From which individual does the milk come from = Write id nr, if many cows write the three most newly calved cows.
Disease = Write what disease or the sympthoms, i.e. arthritis, pneumonia, diarrhoea, cough and so on
Has the calf received treatment = Write any treatment, even rehydration solutions or nsaids, please also note the dose given.

	**Time**	**Volume of milk** (Liter)	**Colostrum** (Mark with X)	**Transition milk** (Mark with X)	**Whole milk** (Mark with X)	**Powder milk** (Mark with X)	**Milk from cow with mastitis** (Mark with X)	**Water was added** (Mark with X)	**From which individual does the milk come from** (id nr)	**Disease** (write symthoms or diagnose)	**Has the calf received treatment?**
**Day 4 Morning**											
**Day 4 Evening**											
**Day 5 Morning**											
**Day 5 Evening**											
**Day 6 Morning**											
**Day 6 Evening**											
**Day 7 Morning**											
**Day 7 Evening**											
