# Supplementary material for: α-Tocopherol and β-carotene concentrations in feed, colostrum, cow and calf serum in Swedish dairy herds with high or low calf mortality
Source: Acta Vet Scand. 2018 Feb 1;60:7. doi: 10.1186/s13028-018-0361-0 (PMC5796441; doi:10.1186/s13028-018-0361-0)
Supplement: Supplementary file 5 — Additional file 5. Regional differences concerning proportion of maize of total diet in 19 Swedish dairy herds with high (n = 9) or low (n = 10) calf mortality risk, day 1–90. [file 13028_2018_361_MOESM5_ESM.docx]

| Additional file 5. Regional differences concerning proportion of maize of total diet in 19 Swedish dairy herds^a^ | | | | |
| --- | --- | --- | --- | --- |
| % maize silage (DM) of total diet | | | | |
| Region | 25th pct. | 50th pct. | 75th pct. | *P*-value^b^ |
| Halland/Skåne | 7 | 17 | 22 |  |
| Skaraborg | 0 | 0 | 17 | 0.0045 |

^a^With high (n=9) or low (n=10) calf mortality risk, day 1-90

^b^Tested with Wilcoxon Rank sum Test
